# Renoprotective, anti-oxidative and anti-apoptotic effects of oral low-dose quercetin in the C57BL/6J model of diabetic nephropathy

**DOI:** 10.1186/1476-511X-13-184

**Published:** 2014-12-06

**Authors:** Isabele BS Gomes, Marcella L Porto, Maria Carmen LFS Santos, Bianca P Campagnaro, Thiago MC Pereira, Silvana S Meyrelles, Elisardo C Vasquez

**Affiliations:** Department of Pharmaceutical Sciences, Health Sciences Center, Federal University of Espirito Santo (UFES), Vitoria, Brazil; Department of Physiological Sciences, Laboratory of Translational Physiology, Health Sciences Center, UFES, Vitoria, Brazil; Pharmaceutical Sciences Graduate Program, Vila Velha University (UVV), Vila Velha, ES Brazil

**Keywords:** Quercetin, Diabetic nephropathy, Oxidative stress, Apoptosis, Flavonoids

## Abstract

**Background:**

Diabetic nephropathy (DN) is one of the major causes of end-stage renal disease in diabetic patients. Increasing evidence from studies in the rodents has suggested that this disease is associated with increased oxidative stress due to hyperglycemia. In the present study, we evaluated the renoprotective, anti-oxidative and anti-apoptotic effects of the flavonoid quercetin in C57BL/6J model of DN.

**Methods:**

DN was induced by streptozotocin (STZ, 100 mg/kg/day, for 3 days) in adult C57BL/6J mice. Six weeks later, mice were divided into the following groups: diabetic mice treated with quercetin (DQ, 10 mg/kg/day, 4 weeks), diabetic mice treated with vehicle (DV) or non-treated non-diabetic (ND) mice.

**Results:**

Quercetin treatment caused a reduction in polyuria (~45%) and glycemia (~35%), abolished the hypertriglyceridemia and had significant effects on renal function including, decreased proteinuria and high plasma levels of uric acid, urea and creatinine, which were accompanied by beneficial effects on the structural changes of the kidney including glomerulosclerosis. Flow cytometry showed a decrease in oxidative stress and apoptosis in DN mice.

**Conclusion:**

Taken together, these data show that quercetin effectively attenuated STZ-induced cytotoxicity in renal tissue. This study provides convincing experimental evidence and perspectives on the renoprotective effects of quercetin in diabetic mice and outlines a novel therapeutic strategy for this flavonoid in the treatment of DN.

## Introduction

Diabetic nephropathy (DN) is one of the most important microvascular complications of diabetes mellitus [1–3] and is the largest single cause of end stage renal disease [4, 5], that leads to a decrease in quality of life and an increased risk of mortality [6]. Recent data indicate that reactive oxygen species (ROS) play a pivotal role in the pathophysiology of DN [7–9]. Persistent hyperglycemia is the main determinant of initiation, promotion and sustentation of DN and it contributes to oxidative stress via two major mechanisms, augmentation of ROS [7, 10–13] and the attenuation of antioxidative mechanisms through glycation of antioxidant enzymes [1, 12, 14].

The development of experimental models of DN has provided a valid approach to characterize its pathogenesis and to create new possibilities for the diagnosis and treatment of this disease. In this regard, the pancreatic islet cell toxin streptozotocin (STZ) has been widely used to induce diabetes in rodents [1, 15, 16], mainly in rat models. Likewise in rat models, the mouse has become attractive for the understanding of human diseases due to the similarities in physiology and to the advantage that its genome can be easily manipulated [17] to produce models of complex genetic diseases such as atherosclerosis [18–20], which is not commonly developed in rats.

Although cumulative evidence suggests that increased oxidative stress may play a crucial role in the pathogenesis of DN [21, 22], antioxidant therapy has shown conflicting results during the treatment of DN in diabetic patients [23]. Currently, only blockers of the renin-angiotensin system are chronically used; however, there are limitations of this mechanism [23, 24] which justifies the search for effective and safer antioxidant candidates. The flavonoids are plant phenolic compounds that exhibit strong antioxidant properties and are widely distributed throughout the plant kingdom; thus, flavonoids are a therapeutic option [25, 26].

Quercetin is a bioflavonoid found in red wine and numerous fruits, vegetables, and nuts [27, 28]. Recently, biochemical and pharmacological studies of quercetin have shown that it is a potent scavenger of ROS and possibly reduces the risk of cardiovascular and renal diseases [1, 28–30]. However, its effects on kidney function, excessive ROS production and kidney cell apoptosis, that are supposed to occur in this mouse model of DN, have not yet been evaluated. Therefore, the present study was designed to test the hypothesis that quercetin presents nephroprotective effects on the main parameters that characterize the DN. We focused our model in the C57BL/6J, which has been the most used genetic background mouse [18–20] but not yet investigated as a model of DN.

## Materials and methods

### Animals

Eight-week-old homozygous C57BL/6J mice were obtained from the Laboratory of Translational Physiology, Health Sciences Center, at the Federal University of Espirito Santo, Brazil. Animals were housed at room temperature (22°C) in a humidity-controlled environment with a 12-h light/12-h dark cycle in the Experimental Unit of the Laboratory of Translational Physiology. Mice were allowed free access to water and a standard laboratory chow diet (Labina®) until experiments were performed. The animals were studied according to the principles devised by the National Institute of Health (NIH) Guide for the Care and Use of Laboratory Animals and the protocol was previously approved by the Institutional Ethics Committee for the Use of Animals (CEUA, Protocol # 013/2010).

### Experimental protocol

Mice were rendered diabetic by intraperitoneal injection of STZ (Boehringer-Mannheim, Mannheim, Germany) diluted in citrate buffer (10 mM, pH 4.5) at a dose of 100 mg/kg/day for 3 days, The control group (non-diabetic, ND, n = 10) was administered an equivalent volume of the vehicle citrate buffer. One week after STZ injection, glycemia was measured after 6 hours of food deprivation and animals with glucose levels greater than 250 mg/dL for at least 2 days were considered diabetic. Six weeks after STZ injections, diabetic mice were randomly divided and received either no treatment (vehicle soy oil, DV, n = 10) or oral quercetin (DQ, n = 10) at a dosage of 10 mg/kg per day for 4 weeks. This dosage was based on previous studies with hypertensive and diabetic animals [31, 32].

### Metabolic and biochemical parameters

After STZ injection, all animals were weighed once a week. At week 4, the animals were housed for a 24-hour period in individual metabolic cages for adaptation. Following this period, a known volume of water and quantify of food were placed in the drinking bottles and the feeder, respectively. After 24 hours, the volume of liquid and chow remaining in the cages were measured. Urine was collected and its volume and protein concentration was determined. Animals were euthanized via an overdose of thiopental (Cristalia, Sao Paulo, Brazil, 200 mg/kg, i.p.) after a period of food deprivation, and their blood was collected for glucose, creatinine, cholesterol, triglycerides, urea and uric acid measurements using colorimetric kits. Proteinuria was determined by a Bradford assay [33]. Tissues were perfused with cold phosphate-buffered saline (PBS, pH 7.4, 0.1 mol/L) through the left ventricle and the kidney was fixed in Duboscq-Brazil solution for histological evaluation.

### Kidney histology

After perfusion, the mouse kidneys were rapidly fixed with Duboscq solution, weighed and processed for morphometric and histological analyses. Samples were dehydrated with a graded series of alcohols, embedded in paraffin, sectioned into 3-μm-thick slices and stained with hematoxylin and eosin for light microscopic morphological studies (AX70, Olympus, Center Valley, PA, USA). Images were captured at a 40x magnification with a color video camera (VKC150, Hitachi, Tokyo, Japan) connected to a microscope (AX70, Olympus, Center Valley, PA, USA). All morphometric and histological analysis were performed in a blinded manner. The mean glomerular tuft area of each kidney was obtained by calculating the mean value of 30 individual glomeruli measured by Image J software (version 1.33u, Public Domain). Masson’s trichrome staining was used to quantify glomerulosclerosis. A total of 30 glomeruli were used to calculate the percentage of the stained area for each kidney using the Image J program (Public Domain Image Processing Program, National Institutes of Health, Bethesda, MD).

### Measurement of intracellular superoxide anions by flow cytometry

Fractions enriched for kidney cell were obtained from each of the groups, were prepared based on previous studies and standardized in our laboratory. The kidney was grossly minced using surgical scissors and was incubated in an isolation solution containing collagenase type II (Gibco Life Technologies, Sao Paulo, SP, Brazil) to dissociate the cells. The cell suspension was filtered through a nylon screen (BD Falcon 70 μm) to remove cellular debris. The samples were then washed twice in PBS before further analysis. Dihydroethidium (DHE, 160 μM) was added to cell suspensions (10^6^ cells) which were then incubated at 37°C for 30 min in the dark in order to determine the intracellular production of superoxide anions (•O_2_^-^) [34–36]. DHE is freely permeable to cells and is rapidly oxidized to ethidium, which binds to DNA and results in the amplification of a red fluorescence signal. Samples were treated with 10 mM doxorubicin for 5 min to induce oxidative stress in the absence of cell toxicity; these samples served as positive control. Cells incubated with ethanol served as the negative control. Cells were then washed, resuspended in PBS, and maintained on ice for immediate detection via flow cytometry (FACSCanto II, Becton Dickinson, San José, CA, USA). Data were analyzed using FACSDiva software (BD Company), and overlay histograms were constructed using FCS Express software. For DHE fluorescence quantification, samples were acquired in duplicate, and 10,000 events were obtained for each measurement. Red fluorescence was detected between 564 and 606 nm using a 585/42 bandpass filter. Data are expressed as the geometric mean fluorescence intensity.

### Apoptosis

Apoptotic kidney cells were quantified via annexin V-fluorescein isothiocyanate (FITC) and propidium iodide (PI) staining, using a commercial detection kit (Becton Dickinson, San José, CA, USA). Briefly, renal cells were washed twice with PBS and the final volume was adjusted to 500 μl with binding buffer (5 × 10^5^ cells). Then, 2 μl of annexin V–FITC and 2 μL of PI were added to the solution and the cells were gently vortexed. Cells were then incubated for 15 min at room temperature (25°C) in the dark. Finally, cells were analyzed using a FACSCanto II flow cytometer (BD). Apoptosis was quantified by analysis of the flow cytometry data after annexin V-FITC and PI labeling.

Kidney cells that were annexin V-FITC^-^/PI^+^ were classified as damaged (Q1). Cells that were annexin V-FITC^+^/PI^+^ were considered to be late apoptotic or secondary apoptotic cells (Q2). Cells that were annexin V-FITC^-^/PI^-^ were considered live cells (Q3). Cells that were annexin V-FITC^+^/PI^-^ were classified as early or primary apoptotic cells (Q4) [37].

### Statistical analysis

All data are expressed as the means ± SEM. The Kolmogorov-Smirnov test showed that variables had a normal (Gaussian) distribution. Flow cytometry data of ROS production are expressed as the geometric MFI–variation coefficient of two repeated and statistically reproducible measurements of at least 5 independent animals (the Friedman test). The statistical analysis was performed by one-way analysis of variance (ANOVA). When the ANOVA showed significant differences, the Tukey’s test was applied as a *post hoc* analysis. The differences were considered significant when p < 0.05.

## Results

### Metabolic parameters

**Figure 1:**
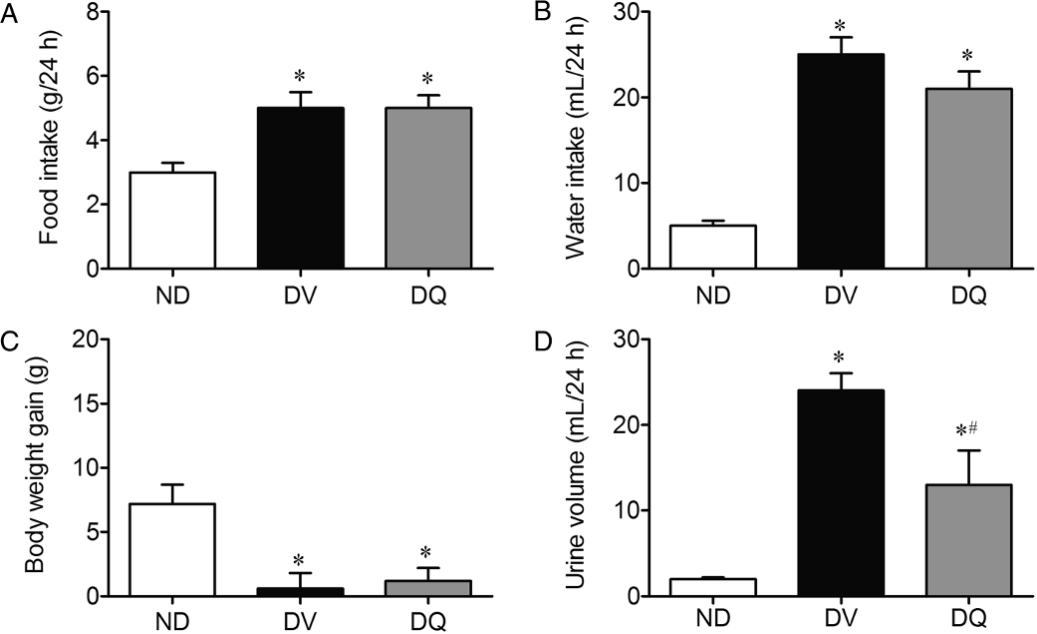
**Food (A) and water (B) intake, body weight gain (C) and urine volume (D) in diabetic mice treated with quercetin (DQ) compared to diabetic mice administered vehicle (DV) and non-diabetic (ND) mice.** Values are means ± SEM for *n =* 6–8 mice per group. *p < 0.05 vs. ND, ^#^ *p <* 0.05 vs. DV.

Figure 1 summarizes the general physical characteristics, diuresis, and food and water intake, 10 weeks after the induction of diabetes. Diabetic mice exhibited hyperphagia (5.0 ± 0.5 g/day, Figure 1A) and polydipsia (25 ± 2 mL/24 h, Figure 1B) when compared to non-diabetic mice (3.0 ± 0.26 g/day and 5.0 ± 0.6 mL/24 h). Quercetin treatment did not show a significant effect on food and water intake. Diabetic mice exhibited a severe polyuria (24 ± 2 mL/24 h, Figure 1D) when compared to non-diabetic mice (1.9 ± 0.2 mL/24 h), and this parameter was reduced by approximately 46% in diabetic mice treated with quercetin. Body weight was similar among the 3 groups; however, over the 2-week period, body weight gain in the non-diabetic group (28% g, p < 0.05) neither the diabetic mice nor the diabetic mice treated with quercetin exhibited significant body weight gain (+0.6 ± 1.2 and +1.2 ± 1.0 g, respectively, Figure 1C).

### Biochemical parameters

**Figure 2:**
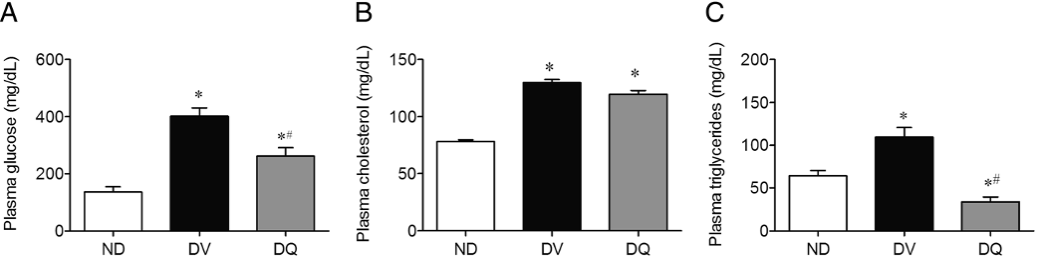
**Plasma glucose (A), total cholesterol (B) and triglycerides (C) in diabetic mice treated with quercetin (DQ) compared to diabetic mice administered vehicle (DV) and non-diabetic (ND) mice.** Values are means ± SEM for *n =* 6–8 mice per group. *p < 0.05 vs. ND, ^#^ *p <* 0.05 vs. DV.

As expected, the results summarized in Figure 2 show that diabetic mice exhibited a significant increase in plasma glucose (3-fold), total cholesterol (1.7-fold) and triglycerides (1.7-fold) levels when compared to non-diabetic mice (135 ± 9 mg/dL, 78 ± 2 mg/dL and 64 ± 6 mg/dL, respectively). Treatment with quercetin caused a significant attenuation of plasma hyperglycemia (35%) and failed to reverse the hypercholesterolemia; however, it diminished the hypertriglyceridemia to levels of 34 ± 6 mg/dL, which was 50% lower than levels observed in the non-diabetic mice.

### Renal function parameters

**Figure 3:**
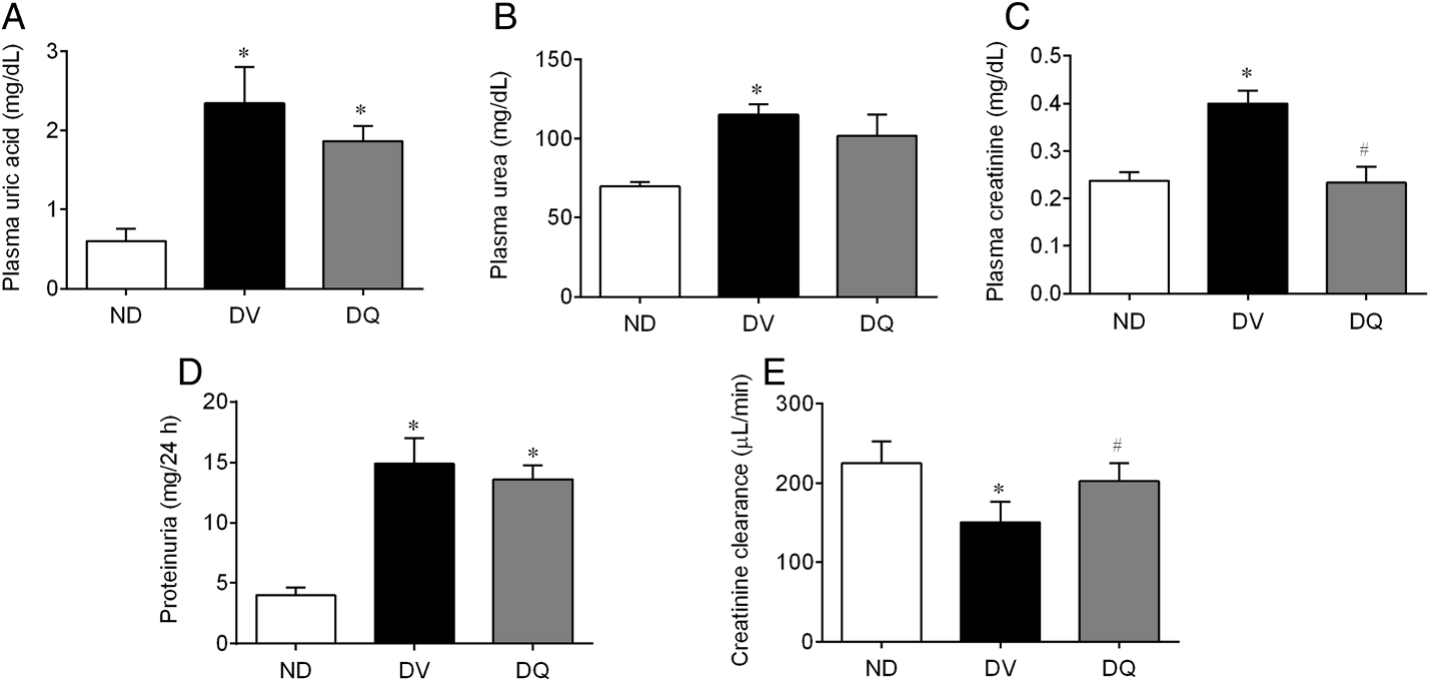
**Plasma uric acid (A), urea (B), creatinine (C), proteinuria (D) and creatinine clearance (E) in diabetic mice treated with quercetin (DQ) compared to diabetic mice administered with vehicle (DV) and to non-diabetic (ND) mice.** Values are means ± SEM for *n =* 6–8 mice per group. *p < 0.05 vs. ND, ^#^ *p <* 0.05 vs. DV.

Figure 3 summarizes the mean values of the parameters used as indices of renal function. Diabetic mice exhibited significantly high plasma concentrations of uric acids (~3-fold, Figure 3A), urea (25%, Figure 3B) and creatinine (42%, Figure 3C) when compared to non-diabetic animals (0.6 ± 0.15 mg/dL, 70 ± 3.00 mg/dL and 0.24 ± 0.02 mg/dL, respectively, p < 0.05). Quercetin showed a tendency to reduce the hyperuricemia and significantly reduced the levels of plasma creatinine and urea levels to values similar to those observed in the non-diabetic group (Figure 3C). Clearance of creatinine was 225 ± 28 μL/min in non-diabetic mice and was significantly reduced in diabetic mice (34%); however, creatinine clearance returned to normal levels upon treatment with quercetin (Figure 3E). Proteinuria, which is another index of renal function, was significantly increased (~3-fold, p < 0.05, Figure 1D) in the diabetic mice when compared to non-diabetic mice (4.0 ± 0.4 mg/24 h, p < 0.05). Treatment with quercetin tended to reduce levels of proteinuria, but these levels were still significantly higher than those observed for non-diabetic mice (Figure 3D).

### Kidney morphometric parameters

**Figure 4:**
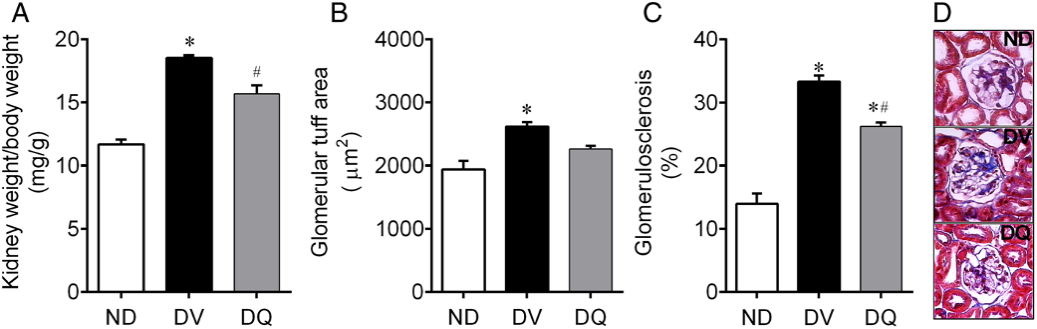
**Kidney weight/body weight ratio (A), glomerular tuff area (B), glomerulosclerosis (C) in diabetic mice treated with quercetin (DQ) compared to diabetic mice administered with vehicle (DV) and non-diabetic (ND) mice.** The mean value of 30 individual glomeruli areas from each kidney were used to calculate the glomerular tuff area and glomerulosclerosis. Micrographs **(D)** are representative glomerular sections (magnification of 400x) stained with Masson trichrome to identify sclerosis (blue) in each glomerulus. Values are means ± SEM for *n =* 6–8 mice per group. *p < 0.05 vs. ND, ^#^ *p <* 0.05 vs. DV.

Diabetes was associated with an increase of approximately 18% in kidney weight/body weight ratio when compared to non-diabetic mice (13.2 ± 0.3 mg/g, p < 0.05), whereas quercetin attenuated this diabetic effect (Figure 4A). The glomerular tuft area analysis of each kidney demonstrated a significant increase of approximately 30% in diabetic mice when compared to non-diabetic mice and quercetin showed a tendency to attenuate this glomerular injury (Figure 4B). As illustrated in the typical photomicrographs (Figure 4D), glomerulosclerosis, appeared more often in the diabetic mice which was characterized by a deposition of extracellular matrix in the mesangium and by glomerular hyperplasia, was more important in the diabetic than in the non-diabetic mice and quercetin had a beneficial effect on this parameter (4C). On average, diabetic animals showed a significant increase in the glomerular tuff area (30%, p < 0.05) and in the occurrence of glomerulosclerosis (~3-fold, p < 0.05) when compared to the non-diabetic mice (2517 ± 124 μm^2^ and 23 ± 1.2%, respectively), and diabetic mice treated with quercetin showed values similar to those observed in non-diabetic mice.

### Oxidative stress

**Figure 5:**
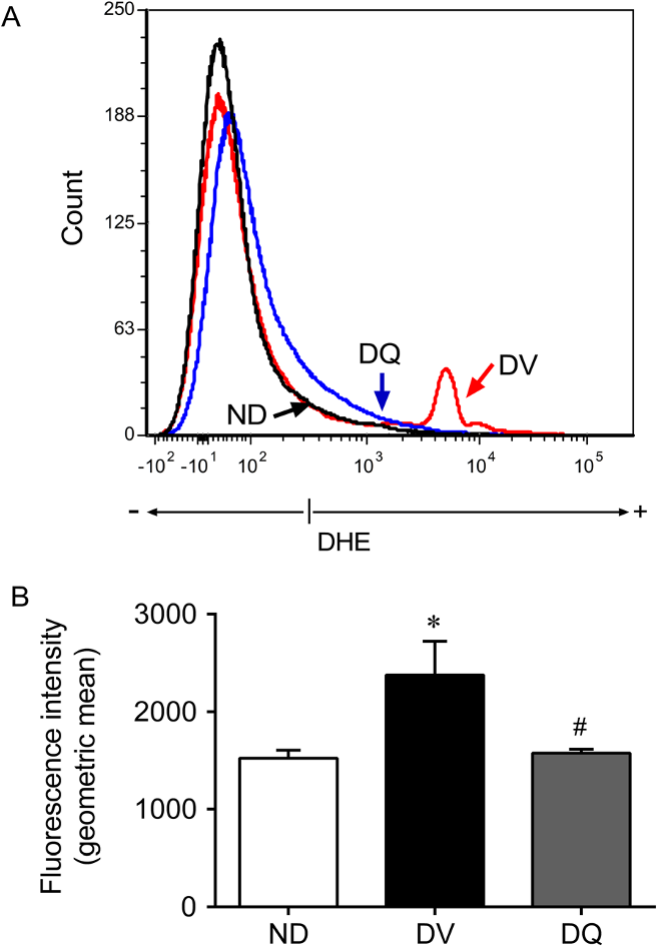
**Production of superoxide anions. A**: representative histograms from flow cytometry analysis using dihydroethidium (DHE) in diabetic mice treated with quercetin (DQ), compared to diabetic mice that received the vehicle (DV) and non-diabetic (ND) mice; the log fluorescence (X-axis) shows the intensity of fluorescence (+) for the number of kidney cells assayed. **B**: bar graph shows geometric mean fluorescence intensity. *p < 0.05 *vs.* ND group, ^#^ *p <* 0.05 *vs.* DV group.

We evaluated ROS production using flow cytometry and DHE to quantify the generation of superoxide anions (•O_2_^-^); the presence of this compound is indicated by the geometric mean fluorescence intensity (in a.u.). Typical histograms obtained via flow cytometry analysis show a rightward-shift in the log DHE fluorescence in diabetic mice (Figure 5A) when compared to non-diabetic mice and diabetic mice treated with quercetin. As summarized in Figure 5B, we observed a remarkable increase in the levels of •O_2_^-^ in DV animals (56%) when compared to ND mice (1524 ± 81 a.u., p < 0.05). Quercetin treatment significantly reduced the levels of oxidative stress to levels similar to those observed in ND mice.

### Apoptosis

**Figure 6:**
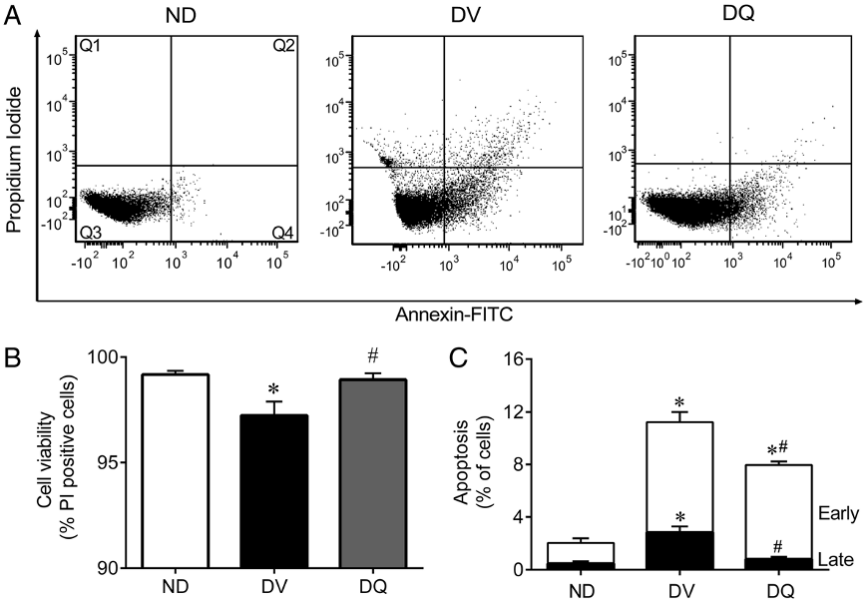
**Flow cytometric analysis of apoptosis in kidney cells.** Each dot plot **(A)** was constructed using propidium iodide (PI) and annexin V/FITC staining indicating: damaged cells (Q1), cells that are undergoing late apoptosis (Q2), viable cells (Q3) and cells in early apoptosis (Q4). Bar graph **B** shows the average percentage of PI positive (viable) cells, which were used to quantify apoptosis. Bar graph **C** shows the average percentage of cells in early and late apoptosis in non-diabetic mice (ND), diabetic mice administered vehicle (DV) and diabetic mice treated with quercetin (DQ). Values are mean ± SEM for 6 to 8 animals per group. *p < 0.05 vs. ND group and ^#^p < 0.05 vs. DV group.

One aim of this study was to evaluate the viability of kidney cells using PI and annexin V staining via flow cytometry analysis. Although the percentage of viable kidney cells was significantly decreased in diabetic mice, greater than 95% of the kidney cells obtained from the three groups of animals were viable which provided excellent conditions for the evaluation of apoptosis (Figure 6B). Typical histograms (Figure 6A) show that DV animals exhibited greater percentage of kidney cells in the early apoptosis quadrant (Q4: annexin V^+^/PI^-^), in the late apoptosis quadrant (Q2: annexin V^+^/PI^+^) and in the damaged cell quadrant (Q1: annexin V^-^/PI^+^). In contrast, the number of cells in the quadrants Q4, Q2 and Q1 was markedly reduced for mice treated with quercetin. Figure 6C summarizes the percentage of cells in early and late apoptosis. Diabetic animals (DV) showed a marked increase in the percentage of cells in early (4-fold) and late apoptosis (6-fold) when compared to non-diabetic (ND) mice (1.58 ± 0.35% and 0.46 ± 0.15%, respectively). Treatment of diabetic (DV) mice with quercetin (DQ) had a marked effect on the progression from early to late apoptosis (Figure 6C). Quercetin treatment tended to reduce the high levels of early apoptosis, but quercetin was able to decrease the percentage values to levels similar to those observed in non-diabetic animals.

## Discussion

This work showed that chronic oral treatment with low-dose quercetin exerts antidiabetic effects and attenuates the development of nephropathy in STZ-induced DN mice. These results are supported by a decrease of plasma glucose, creatinine, triglycerides, proteinuria and diminution of mesangial matrix expansion accompanied by a reduction in •O_2_^-^ production and apoptosis in kidney cells.

STZ-injected mice exhibit destruction of β cells and reduction in insulin secretory capacity [15, 38]. Consequently, these mice develop typical characteristics of diabetes such as polydipsia, polyuria, and proteinuria accompanied by loss of body weight (even with polyphagia) as observed in the present study. Furthermore, in accordance with our biochemical and morphological data, we considered the five stages of clinical classification of DN [39, 40] and suggest that this experimental murine model corresponds to a stage 4 clinical classification due to its declining glomerular filtration rate and proteinuria; thus, this model is suitable for evaluating the nephroprotective effects of the bioflavonoid quercetin. It must be noted that we avoided interfering with the intrinsic nephrotoxic effects of STZ by acquiring our data 6 weeks after STZ administration when the kidneys are known to have recovered from the acute renal injury caused by STZ [38, 41, 42]; thus, we can assume that the damages observed in our model was due only to diabetic status.

Interest in studies of the effects of natural antioxidants prevent oxidative damage in mouse models of diabetes has recently grown [2, 38, 43]. The best candidates are generally molecules that show high power antioxidant capacity, high permeability to mitochondrion [44], long half-lives [45] and enhancement of enzyme activity [43]; quercetin exhibits all of these traits. This bioflavonoid may inhibit the formation of ROS in the three different ways: (a) by interacting directly with •O_2_^-^ during initiation, (b) by forming hydroxyl radicals via the chelation of iron ions, and (c) by directly reacting with lipid peroxyl radicals scavengers during lipid peroxidation [46]. Additionally, it is possible that quercetin increases the activity of superoxide dismutase, catalase, glutathione peroxidase, glutathione reductase and glutathione [43, 47]. All these effects may promote radical scavenging activity, anti-inflammatory and anti-apoptotic effects and contribute to renal protection against STZ-induced DN.

Recent data has shown that the persistent hyperglycemia leads to an increase in the activity of several pathways involved in the disease that contribute to oxidative stress: (1) auto-oxidation of glucose, (2) advanced glycation end-product (AGE) formation, (3) polyol pathway flux, (4) protein kinase C (PKC) isoforms activation and (5) mitochondrial dysfunction [5, 10, 48–50]. Our results demonstrate that quercetin attenuates hyperglycemia in accordance with some authors [46, 51–54], however, other authors disagree [1, 43]. Independent of this apparent inconsistence, the impact on glycemia may be due to perturbations of some pathways. Previous studies have shown that this bioflavonoid can protect pancreatic β-cells from oxidative stress and damage (either directly or indirectly) and improve insulin secretion in STZ models [51, 53]. Additionally, quercetin can stimulate glucose uptake in peripheral tissues via the translocation of GLUT4 [46, 55, 56] and can increase hepatic glucokinase activity [53], thus augmenting both oxidation and storage of glucose and reducing hepatic gluconeogenesis and glycogenolysis [57]. Moreover, it has been reported that quercetin can blockade α-glucosidase activity *in vitro* [57, 58] and *in vivo* [53], thus inhibiting the digestion and absorption of carbohydrates in the small intestine [59]. In our study, this latter hypothesis provide a better explanation for the hypoglycemic effect exhibited by quercetin, considering that it exhibits low bioavailability via oral administration due to poor solubility and stability [25]. Therefore, we cannot discard the notion that indirect effects of quercetin on oxidative stress are responsible for the improvement in glycemic homeostasis, as recently observed in a mouse model by Alam et al. [56].

It has been shown that decreases in body weight are due to intense dehydration and catabolism of fats and proteins, even under the polyphagia and polydipsia evident conditions observed in the DN group [43, 60]. This hypothesis is supported by the observation that hypertriglyceridemia and, in part, azotemia occurs in diabetic animals. Quercetin treatment was capable of reducing polyuria and fat and protein levels, likely as a consequence of better control of the glycemic state which leads to lower release of fatty acids from adipose tissue and normalizes triglycerides plasma levels without modifying the hypercholesterolemia; these results are similar to those observed by others [11]. It should be noted that, since this flavonoid may decrease LDL and increase HDL [61, 62], we cannot discard the possibility of alteration in the ratio HDL/LDL, maintaining the hypercholesterolemia invariable. Moreover, the dose we used in the present study (10 mg/Kg) was unable to improve body weight, which conflicts with another report [43]. This apparent contradiction could be due to different types of experimental rodent models, the use of higher doses (from 2.5- to 10-fold) or the route of administration (e.g., intraperitoneal), which could increase the bioavailability of quercetin as observed by others [38, 63–65].

Our data corroborate the hypothesis that links renal dysfunction to renal oxidative stress and is in agreement with other reports [1, 56]. Under pathologic conditions of oxidative stress, the increased production of ROS could compromise NO bioavailability and promote the formation of a variety of vasoconstrictive mediators that could affect renal functions, such as tissue perfusion and glomerular filtration [1, 24, 42]. These changes could contribute to increases in serum creatinine, urea and uric acid, accompanied by a reduction of creatinine clearance and proteinuria, as observed by us and by others [66, 67]. It is likely that the antioxidative properties of quercetin decreases the production of vasoactive autacoids, which may play a role in its improvement of renal dysfunction in diabetes. This hypothesis is further supported by reports that quercetin produce a direct vasorelaxant effect in vascular tissues [68–70]. Furthermore, the glomerulus is considerably more sensitive to oxidative injuries than other nephron segments [70]. This and the above factors could explain our finding regarding the improvement in creatinine clearance in the DN group treated with quercetin, despite the absence of the normalization of other renal parameters. It should be emphasized that urea and uric acid can be generated by amino acid and purine catabolism, respectively, and that both products are also excreted by tubular secretion [66, 67].

In addition to the biochemical parameters, our morphological data also demonstrate that quercetin exhibits a nephroprotective effect. In diabetic mice, we observed a significant increase in glomerular tuft size and glomerulosclerosis indicating an early diabetes-induced renal injury; these results are in agreement with other reports [5, 21, 32, 67, 71]. The novelty of the present study is that treatment with quercetin resulted in a significant amelioration of the observed glomerulosclerosis and the kidney weight/body weight ratio, most likely due to the hypoglycemic effect discussed above in addition to its direct antioxidative properties. Oxidative stress plays a major role in the disruption of cellular functions in the kidney and leads to increased vascular permeability and tissue damage [6]. This hypothesis is supported by the finding of others [11, 43] showing evidence of renal oxidative stress in diabetic rats [38, 63–65].

In support of this oxidative stress hypothesis, flow cytometry assessment of dihydroethidium (DHE) fluorescence showed that the increased levels of •O_2_^-^ in the kidneys of diabetic mice was reduced by quercetin treatment and resulted in the abolishment of ROS (Figure 5). This finding corroborates the concept that the overproduction of •O_2_^-^ is implicated in the pathophysiology of diabetic nephropathy and that this flavonoid is a potent scavenger of ROS that could potentially reduce the risk of related diseases [29, 30, 46, 54, 56, 72, 73]. Moreover, oxidative stress is recognized as a strong mediator of apoptosis [43, 54, 74]. Our flow cytometry data show, for the first time that DN in this mouse model is accompanied by apoptosis, indicating a marked effect of this disease on kidney cell function. Interestingly, quercetin treatment was able to greatly ameliorate the onset of both early and late apoptosis in diabetic mice and to restore cell viability. Our data corroborate the findings of Liu et al. [75] who observed lower ROS production and apoptosis in kidneys damaged by lead when treated with the same bioflavonoid; these results are in agreement with others who reported that quercetin possesses anti-apoptotic properties [54, 76, 77]. In contrast, high levels of quercetin increased the number of dead kidney cells, indicating the presence of dose-dependent auto-oxidative activity followed by inhibition of mitochondrial respiration which affect ROS production [43, 78]. Therefore, our data contribute to a better understanding about the potential use of quercetin as an antioxidant in a dose-dependent manner.

Although other studies have tested the effects of quercetin on the DN rat model, it should be considered that the common route of administration has been via intraperitoneal [43, 46, 54, 63]. On the other hand when quercetin is administered via oral [38, 64, 65], the dose has been much higher (25 to 100 mg/Kg) than that we used in the genetic background C57BL/6J mouse. The importance of our data is that we found beneficial effects, including a decreased oxidative stress and anti-apoptotic effect, both detected by direct measurements, even using a lowest relative bioavailability oral dose of quercetin (10 mg/Kg). Thus, these additional data highlight the importance of quercetin as potential nutraceutical for management of DN.

In conclusion, our results were obtained from the most commonly used mouse genetic background (C57BL/6J) and suggest that oxidative stress plays a pivotal role in the pathophysiology of DN and that oral low-dose quercetin exhibits a beneficial effect by ameliorating the consequences of hyperglycemia-induced ROS overproduction in the kidney. Thus, quercetin is a promising therapeutic agent that could potentially be used for the prevention and/or treatment of renal dysfunction caused by diabetes.
